# Identification and characterization of melon circular RNAs involved in powdery mildew responses through comparative transcriptome analysis

**DOI:** 10.7717/peerj.11216

**Published:** 2021-04-15

**Authors:** Jianlei Sun, Yumei Dong, Chongqi Wang, Shouhua Xiao, Zigao Jiao, Chao Gao

**Affiliations:** Shandong Key Laboratory of Greenhouse Vegetable Biology, Shandong Branch of National Improvement Center for Vegetable, Vegetable Science Observation and Experiment Station in Huang huai District of Ministry of Agriculture (Shandong), Institute of Vegetables and Flowers, Shandong Academy of Agricultural Sciences, Jinan, China

**Keywords:** Melon, Powdery mildew disease, Comparative transcriptome, Circular RNA, Expression pattern

## Abstract

Circular RNAs (circRNAs) are a class of newly discovered non-coding RNAs that are typically derived from a genome’s exonic, intronic, and intergenic regions. Recent studies of circRNAs in animals and plants have shown that circRNAs are vital in response to various abiotic and biotic stresses. Powdery mildew disease (PM) is a serious fungal disease threatening the melon industry. We performed whole transcriptome sequencing using the leaves of a PM-resistant (M1) and a PM-susceptible (B29) melon to identify circRNAs and determine their molecular functions. A total of 303 circRNAs were identified and >50% circRNAs were derived from exonic regions. Expression levels were significantly altered in 17 and 23 circRNAs after PM infections in B29 and M1, respectively. Melon circRNAs may participate in the response to biotic stimuli, oxidation reduction, metabolic processes, and the regulation of gene expression based on the functional annotation of circRNA parental genes. Furthermore, 27 circRNAs were predicted to be potential targets or ‘sponges’ for 18 microRNAs (miRNAs). Our results are the first to identify and characterize circRNA functions in melon and may contribute to a better understanding of the role and regulatory mechanisms of circRNAs in resisting PM.

## Introduction

Non-coding RNAs (ncRNAs), including microRNA (miRNA), small interfering RNA (siRNA), circular RNA (circRNA), and long noncoding RNA (lncRNA), account for a large portion of the transcriptome sequences in eukaryotic organisms. They have been shown to play important roles in the regulation of protein coding gene expression at transcriptional and post-transcriptional levels (Ponting, Oliver & Reik, 2009; Djebali et al., 2012; Axtell, 2013). CircRNAs are a class of newly discovered endogenous non-coding RNAs characterized by the formation of a closed loop structure, which can be classified as exonic circRNA, intronic circRNA, or intergenic circRNA according to its origin and position in the genome (Zhang et al., 2013a; Zhang et al., 2013b; Jeck & Sharpless, 2014; Suzuki & Tsukahara, 2014; Chen, 2016). Recent studies indicate that circRNAs are much more stable than linear RNAs and often present distinct expression patterns in specific cell, tissue, and at specific developmental stages (Salzman et al., 2013; Jeck & Sharpless, 2014).

CircRNAs have been studied in humans, mice, mosquitos, and sheep (Salzman et al., 2012; Gruner et al., 2016; Jiang et al., 2014; Li et al., 2017) with little attention given to plant circRNAs. The rapid development of deep-sequencing technology and bioinformatics has led to more circRNAs reported in plants including *Arabidopsis*, rice, wheat, barley, maize, soybean, cotton, and tomato (Dou et al., 2017; Lu et al., 2015; Wang et al., 2017a; Wang et al., 2017b; Darbani, Noeparvar & Borg, 2016; Chen et al., 2018; Zhao et al., 2017; Xiang et al., 2018; Yin et al., 2018). Many circRNAs have been functionally characterized in model species such as *Arabidopsis*, rice, and tomato, suggesting that circRNAs play vital roles in multiple biological processes by regulating the expression of their parental genes or acting as miRNA ‘sponges’ to affect the accumulation of target mRNAs. Liu et al. (2017) identified 6,012 circRNAs using publicly available RNA-seq data from *Arabidopsis* leaves and discovered the function of circRNA in the regulation of the development and senescence of leaves. Tomato is the model plant for studying fruit ripening. Over-expression of a ripening-related circRNA generated from *Phytoene Synthase* 1 (*PSY*1) in tomato leads to a significant decrease of *PSY*1 mRNA abundance and lycopene content (Tan et al., 2017).

CircRNAs have been shown to play important roles in response to various environmental stresses, including biotic and abiotic stresses. Many circRNAs in maize responded to viral infections and phosphate-starvation conditions (Ye et al., 2015; Ghorbani et al., 2018). In wheat, circRNAs can act as ‘sponges’ of corresponding miRNAs to increase resistance to dehydration in wheat seedlings (Wang et al., 2017a; Wang et al., 2017b). Furthermore, 1934 circRNAs in cucumber responded to salt stress. The functional annotation of parental genes revealed that circRNAs may respond to salt stress by mediating signal transcription, metabolism adaptation, and cell cycle and ion homeostasis-related pathways (Zhu et al., 2019). These studies revealed that circRNAs are essential in plant growth and development and aid in their resistance against various environmental stresses. However, information about circRNA has not been reported in melon and little is known about the function of circRNA in resisting powdery mildew disease (PM).

Melon (*Cucumis melo* L.) is an important fruit crop and is very vulnerable to PM in its later developmental stages. PM can decrease fruit yield and quality and has restricted the development of the melon industry worldwide (Zhang et al., 2013a; Zhang et al., 2013b). In the present study, we selected a PM-resistant and a PM-susceptible cultivated melon to study circRNAs. Comparative transcriptome analysis was performed to identify differentially expressed circRNAs. The parental genes of differentially expressed circRNAs were functionally characterized, which provided a foundation for further analysis of the functional and regulatory mechanisms of circRNAs in resisting PM.

## Materials and Methods

### Plant materials and pathogenic fungus infection

Plant materials were collected from melon seedlings with a highly resistant genotype (M1) and a highly susceptible genotype (B29) to powdery mildew fungus. Melons were grown in a greenhouse on an experimental farm at a temperature of 28 °C/20 °C (day period/night period) and a photoperiod of 16/8 h (day/night). Powdery mildew fungus (*Podosphaera xanthii*, *race 1*) was collected from the leaves of cultivated melons and was inoculated onto melon seedlings with two or three true leaves at a concentration of 1 × 10^6^ spores/mL, as previously described (Cohen, 1993). The control seedlings were treated with water. The leaves of M1 and B29 treated with water and powdery mildew fungus were harvested at 24 h and 48 h post-inoculation (identified as mock, M24, M48, B24, and B48, respectively). Three leaves were collected from independent seedlings and pooled as a single biological replicate. Three individual biological replicates were prepared for each treatment and were stored at −80 °C for the experiments.

### Total RNA extraction and library construction

Trizol reagent was used to extract total RNA from all samples according to the manufacturer’s instructions (Invitrogen, CA, USA). The concentration and quality of RNA was measured using a NanoPhotometer spectrophotometer (IMPLEN, CA, USA) and Bioanalyzer 2100 system (Agilent Technologies, CA, USA). RNA integrity was checked using 1% agarose gel. Three µg RNA per sample was used as input material to construct the library. Ribosomal RNAs were removed using the rRNA Removal Kit (Epicentre, WI, USA) following the manufacturer’s instructions, and linear RNAs were digested using RNase R (Epicentre, WI, USA) at 37 °C for one hour. The remaining RNAs were used as templates for reverse transcription in accordance with the manufacturer’s instructions for the RNA-Seq sample preparation kit (Illumina, San Diego, USA). All libraries were sequenced on the Illumina Hiseq 4000 platform (Novogene, Tianjin, China) with a 150 bp paired-end strand-specific sequencing method.

### Bioinformatics analysis and identification of circRNA

To identify circRNA, the raw reads were first filtered using the Fastx-toolkit pipeline (http://hannonlab.cshl.edu/fastx_toolkit/) to remove low-quality reads and to trim the adapter sequences. All clean reads were mapped to the melon reference genome (http://cucurbitgenomics.org/organism/18) using Bowtie2 (v2.2.8; http://bowtie-bio.sourceforge.net/bowtie2/index.shtml), and only uniquely mapped reads with no more than two mismatches were retained for further analysis. We used find_circ (v1.2, https://github.com/marvin-jens/find_circ) and CIRI2 (https://sourceforge.net/projects/ciri/) to analyze and identify the candidate circRNAs with the default parameters (Gao, Zhang & Zhao, 2018).

### Experimental validation of circRNAs

Total RNAs were extracted using Trizol reagent and treated with DNase I at 37 °C for 30 min according to the manufacturer’s instructions (Invitrogen, CA, USA). Genomic DNA (gDNA) was isolated using the Plant DNA Mini Kit (TRANSGEN BIOTECH, Beijing, China). A total of 50 µg DNase I-treated RNA was treated with RNase R for 40 min to remove the rRNA. The first-strand cDNA was synthesized from 2 µg of RNase R-treated RNA with random primers using the cDNA Synthesis SuperMix (TRANSGEN BIOTECH, Beijing, China). PCR amplification was conducted using divergent primers designed on the flanking sequences of head-to-tail splicing sites of circRNAs to validate the head-to-tail back-spliced site of circRNAs in melon. Sanger sequencing was performed to further confirm the presence of the back-spliced junction sites. The divergent primers used for circRNA validation are listed in Table S1.

### Quantification of circRNAs and differential expression analysis

Transcript per million mapped reads (TPM) values were calculated to obtain the expression quantity of all circRNAs. TPM values were calculated based on the read counts mapped to the circRNA. DESeq2 R package (version 2.14, http://www.bioconductor.org/packages/release/bioc/html/DESeq.html) was used for differential expression analysis of circRNAs based on the negative binomial distribution model and the *P*-values were adjusted using Benjamini and Hochberg’s approach to control the false discovery rate. circRNAs with differential expression levels were identified using a criterion of adjusted *P*-values < 0.05 and —log_2_ fold change— ≥ 1.

### qRT-PCR validation of differentially expressed circRNAs

Total RNAs were extracted from melon leaves using a Trizol reagent and were treated with DNase I to digest genomic DNA according to the manufacturer’s instructions (Invitrogen, CA, USA). A total of 50 µg DNase I-treated RNA was treated with RNase R for 40 min to remove rRNA. The first-strand cDNA was synthesized from 2 µg of RNase R-treated RNA with random primers using the cDNA Synthesis SuperMix (TRANSGEN BIOTECH, Beijing, China). qRT-PCR was performed to access the relative expression of circRNA using SYBR Green Master Mix (Bio-Rad, Hercules, California). The qRT-PCR procedure was as follows: 95 °C for 5 min, followed by 40 cycles of 95 °C for 5 s, 58 °C for 30 s, and 72 °C for 30 s on an ABI 7500 Real Time PCR system (Applied Biosystems, Waltham, Massachusetts). *CmActin* was used as an internal reference gene. The 2^−^^△△^^Ct^ method was used to calculate the relative expression quantity with three biological replicates and Duncan’s multiple range test was performed to assess whether the results were statistically different (*P* < 0.01). Primer sequences used in qRT-PCR experiments are listed in Table S1.

### Functional annotation of circRNA parental genes

The genes producing circRNAs were defined as parental genes. Gene Ontology (GO) enrichment analysis of parental genes of differentially expressed circRNAs was carried out using the GOseq R package (https://bioconductor.org/packages/release/bioc/html/goseq.html), and GO terms with adjusted *P*-value < 0.05 were considered to be significantly enriched.

## Results

### Characterization of the phenotype and resistance against PM of M1 and B29

**Figure 1:**
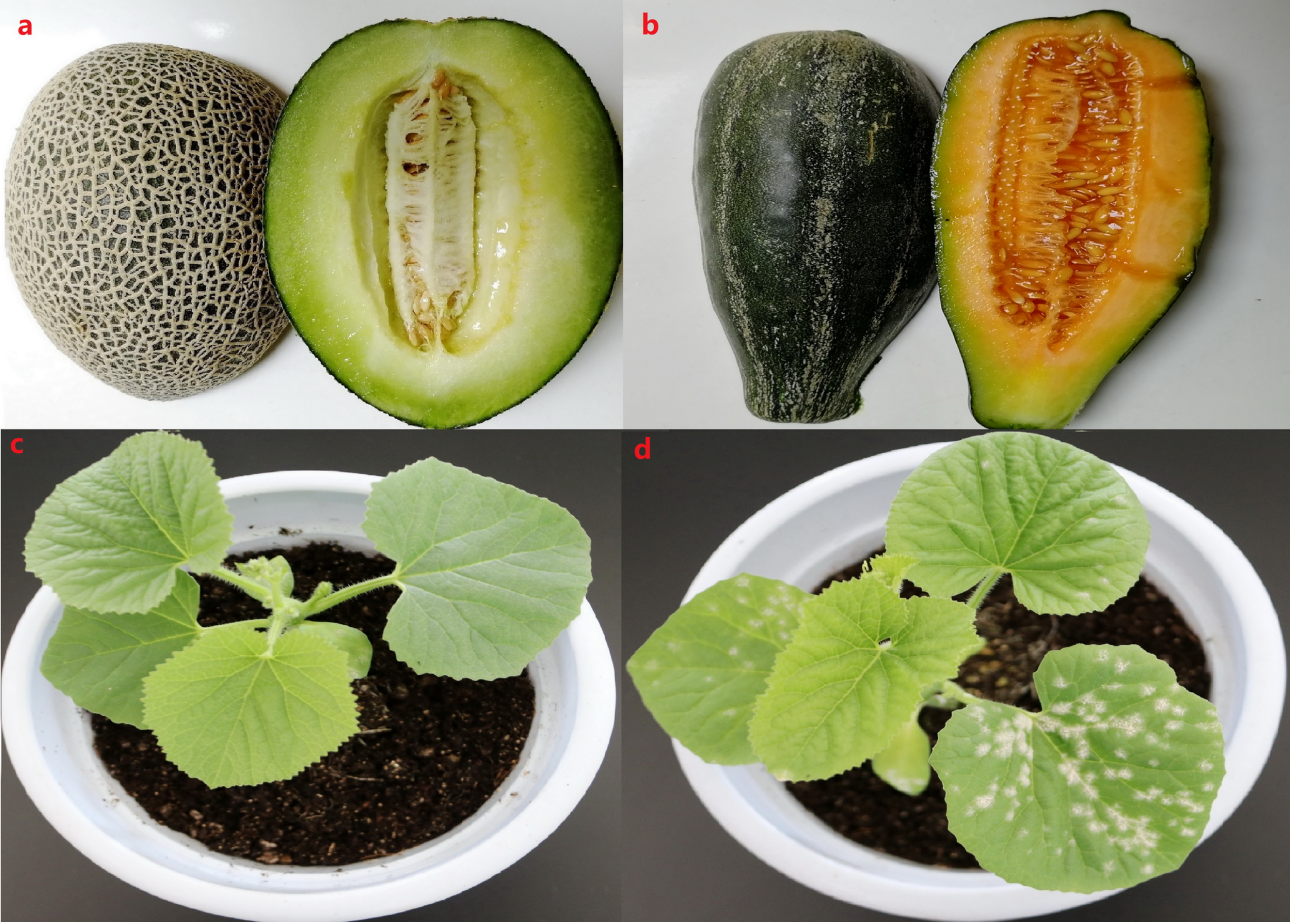
The different phenotype of fruit and leaves observed at 7 day after powdery mildew infection. (A) The phenotype of M1 fruit with thick rind and high net density. (B) The phenotype of B29 fruit with thin and smooth rind. (C) The phenotype of M1 after powdery mildew infection. (D) The phenotype of B29 after powdery mildew infection.

To explore the mechanism underlying PM resistance in melon at the transcriptome level, high-throughput sequencing and comparative transcriptome analysis were performed between genotypes that were highly resistant (M1) and a highly susceptible (B29) to PM fungus. M1 is a homozygous inbred line with a thick rind and high net density that has been self-pollinating for thirteen generations (Fig. 1A). B29 is a homozygous inbred line, distinct from a cultivated melon, with a smooth, thin rind (Fig. 1B). The genetic background of both lines is highly stable and homozygous. The PM resistance of these two inbred lines was confirmed when we observed the amount of bacterial plaque on the leaves after PM fungus inoculation in the greenhouse. Our results showed different symptoms on the leaves of M1 and B29 7 days after PM infection. No obvious bacterial plaque was observed on the leaves of M1 seven days after PM infection (Fig. 1C), suggesting that M1 is a highly resistant genotype. However, the B29 leaves showed an intense bacterial plaque (Fig. 1D), indicating that B29 is a highly susceptible genotype to PM fungus.

### Identification and characterization of circRNAs in melon

**Figure 2:**
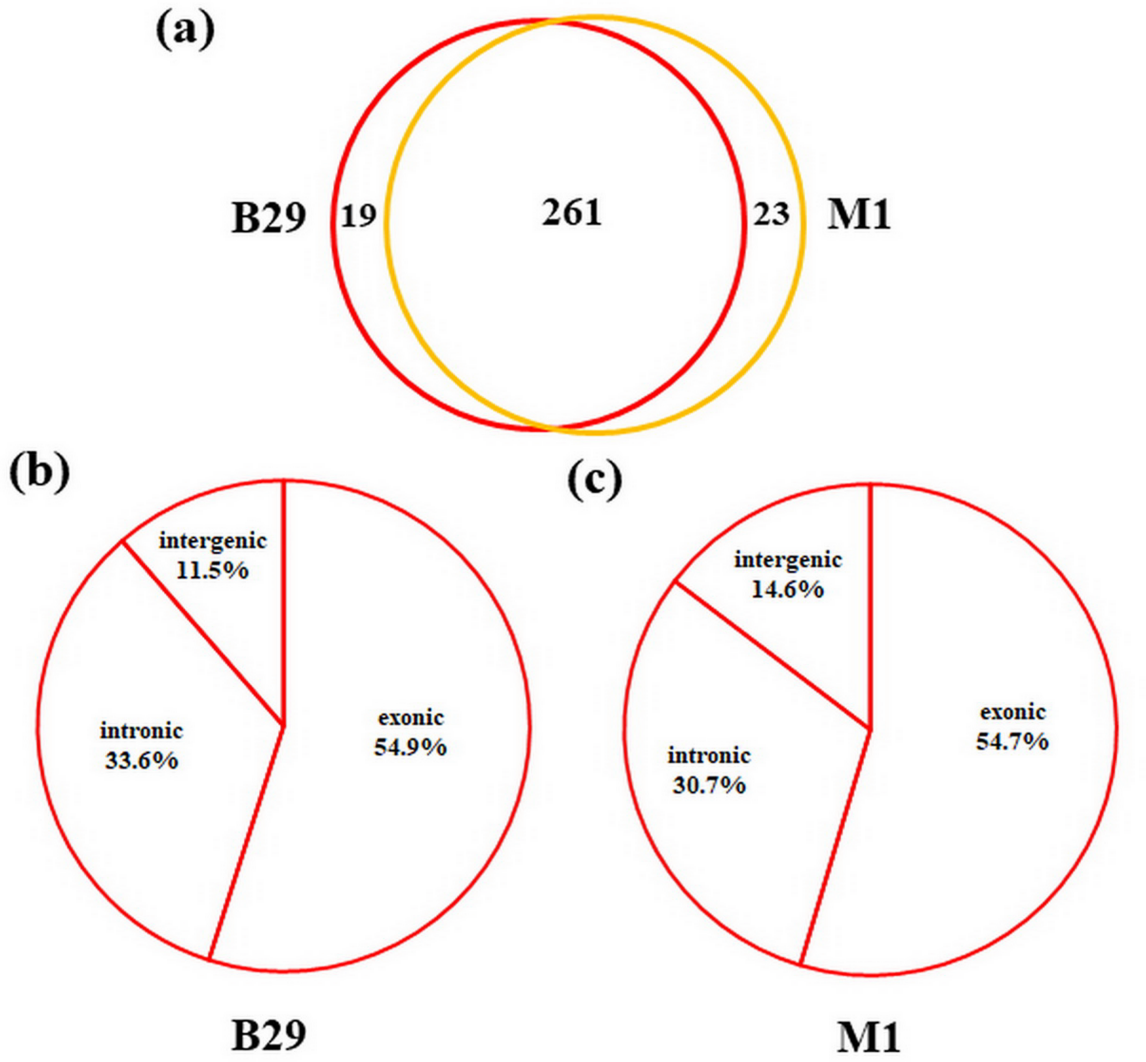
Characterization of melon circRNAs. (A) The number of specific and shared circRNAs in B29 and M1. (B) The percentage of circRNAs derived from intergenic, exon, and intron regions in B29. (C) The percentage of circRNAs derived from intergenic, exon, and intron regions in M1.

**Figure 3:**
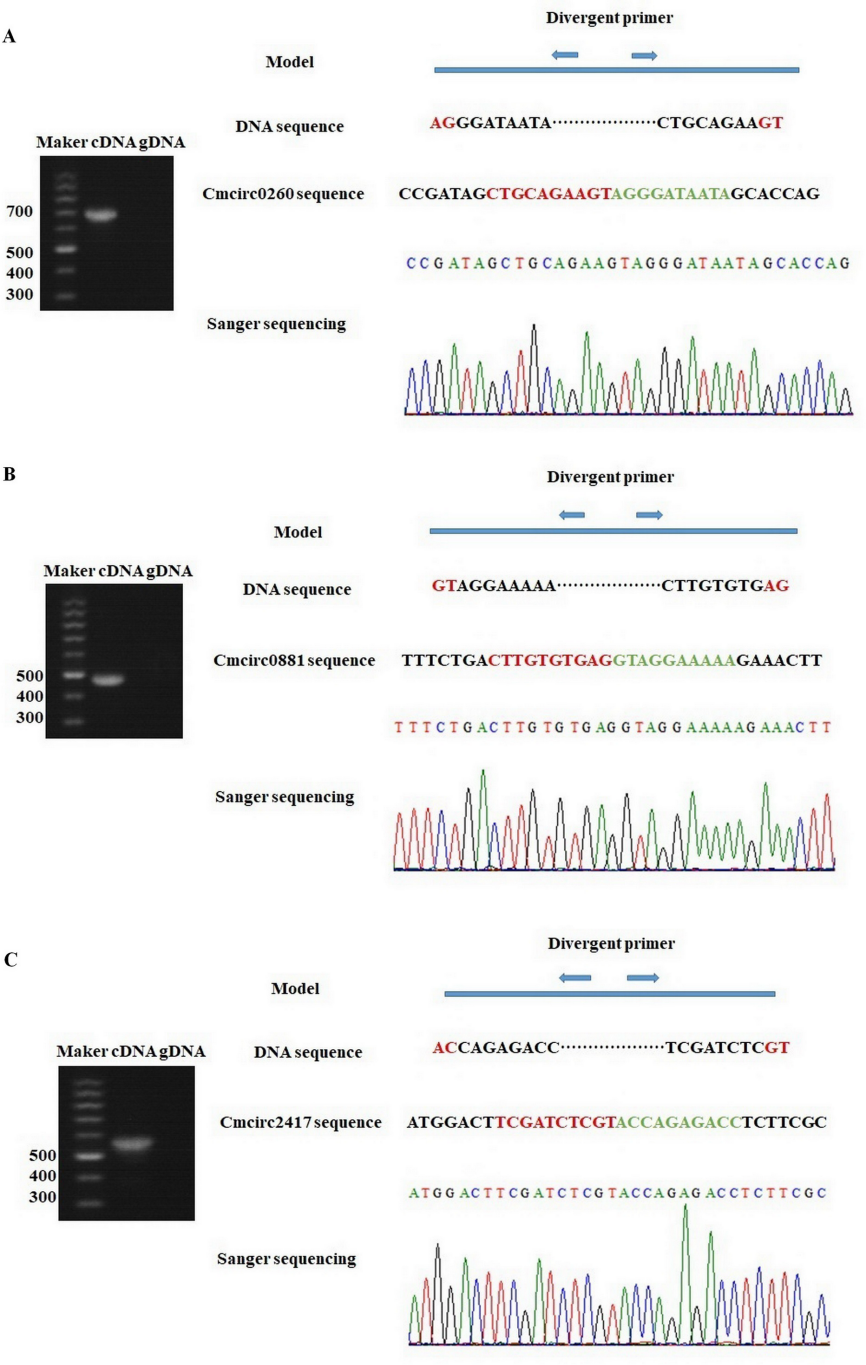
Experimentally validation of Cmcirc0260 (A), Cmcirc0881 (B) and Cmcirc2417 (C). Divergent primers were used for amplification of the cDNA and gDNA. Agarose gel electrophoresis and Sanger sequencing were performed to examine the size and sequence of PCR product.

To detect circRNAs and explore their functions in resisting against PM disease, 18 rRNA-depleted libraries were generated from the leaves of PM-resistant lines and PM-susceptible lines under both control and PM infected conditions, yielding approximately 100 million reads for each library (Table S2). After the low-quality reads were removed, clean reads were mapped to the reference genome and approximately 83% to 86% of clean reads were uniquely mapped to the melon reference genome in each sample (Table S2). A total of 303 circRNAs were identified using the circRNA identification tool find_circ and CIRI2 a. M1 and B29 shared 261 circRNAs, and only 23 and 19 unique circRNAs were specifically accumulated in M1 and B29, respectively (Fig. 2A). CircRNAs were classified into three groups based on their genomic location and orientation, namely exonic circRNAs, intergenic circRNAs, and intronic circRNAs. Interestingly, in both B29 and M1 melon, exonic circRNAs predominated (54.9% and 54.7%, respectively) compared to intergenic and intronic circRNAs (Figs. 2B and 2C). This was consistent with the conclusion that circRNAs are mainly generated from coding regions in both monocot and dicot plants (Lu et al., 2015; Dou et al., 2017). The length of the circRNAs in melon ranged from 150 to 83,568 nt, but most (86.8%) were <2,000 nt (Fig. S1). To validate the head-to-tail back-spliced site of circRNAs identified in melon, divergent primers were designed for three circRNAs to perform PCR amplification. The PCR products were further analyzed by agarose gel electrophoresis and Sanger sequencing. The results showed that all circRNAs had expected size and validated back-spliced junction sites (Fig. 3), indicating that these circRNA were credible.

### Differential expression of circRNAs in response to PM infection and qRT-PCR validation

**Figure 4:**
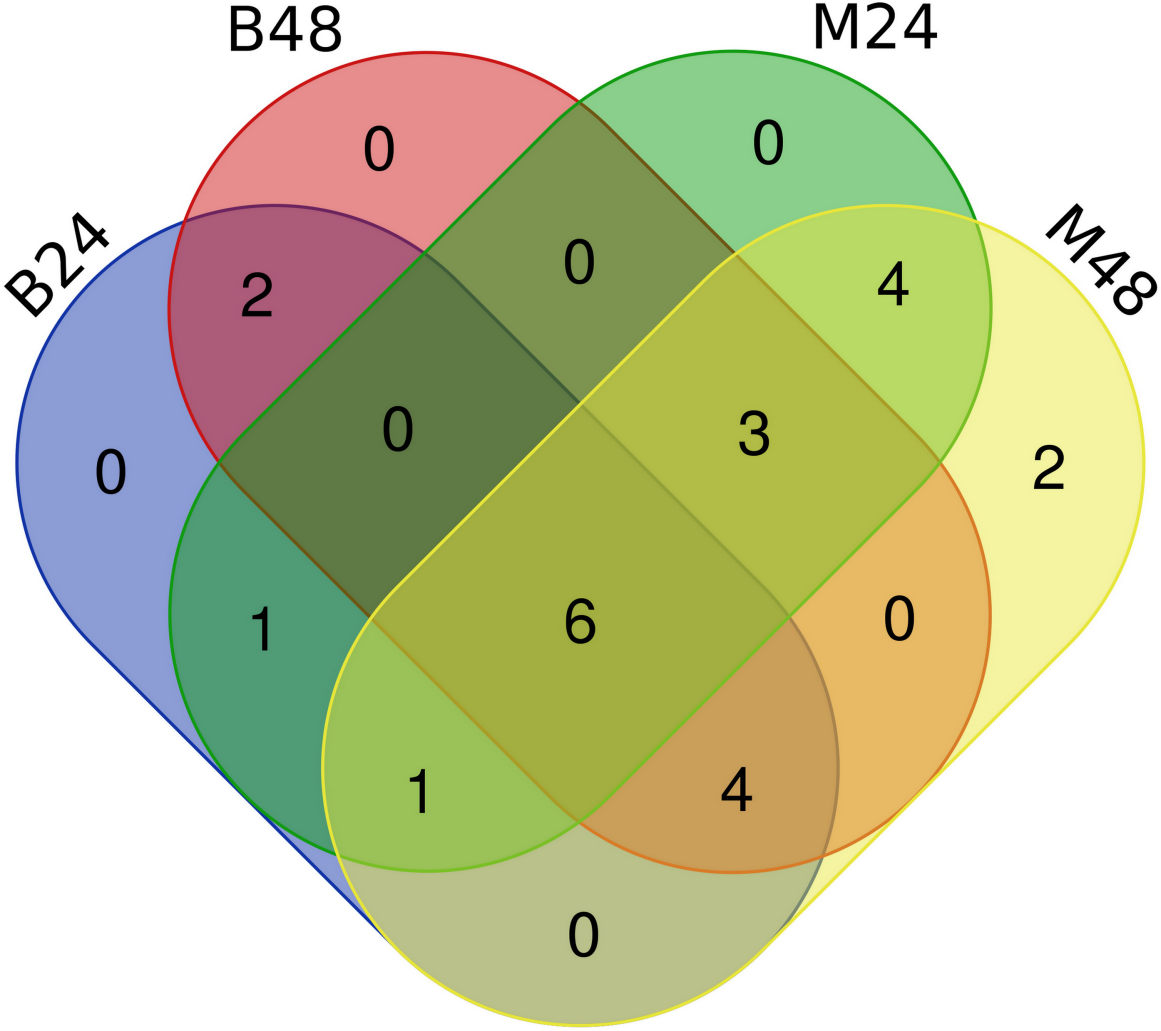
Statistical analysis of differential expressed circRNAs between PM-susceptible melon (B29) and PM-resistant melon (M1).

**Figure 5:**
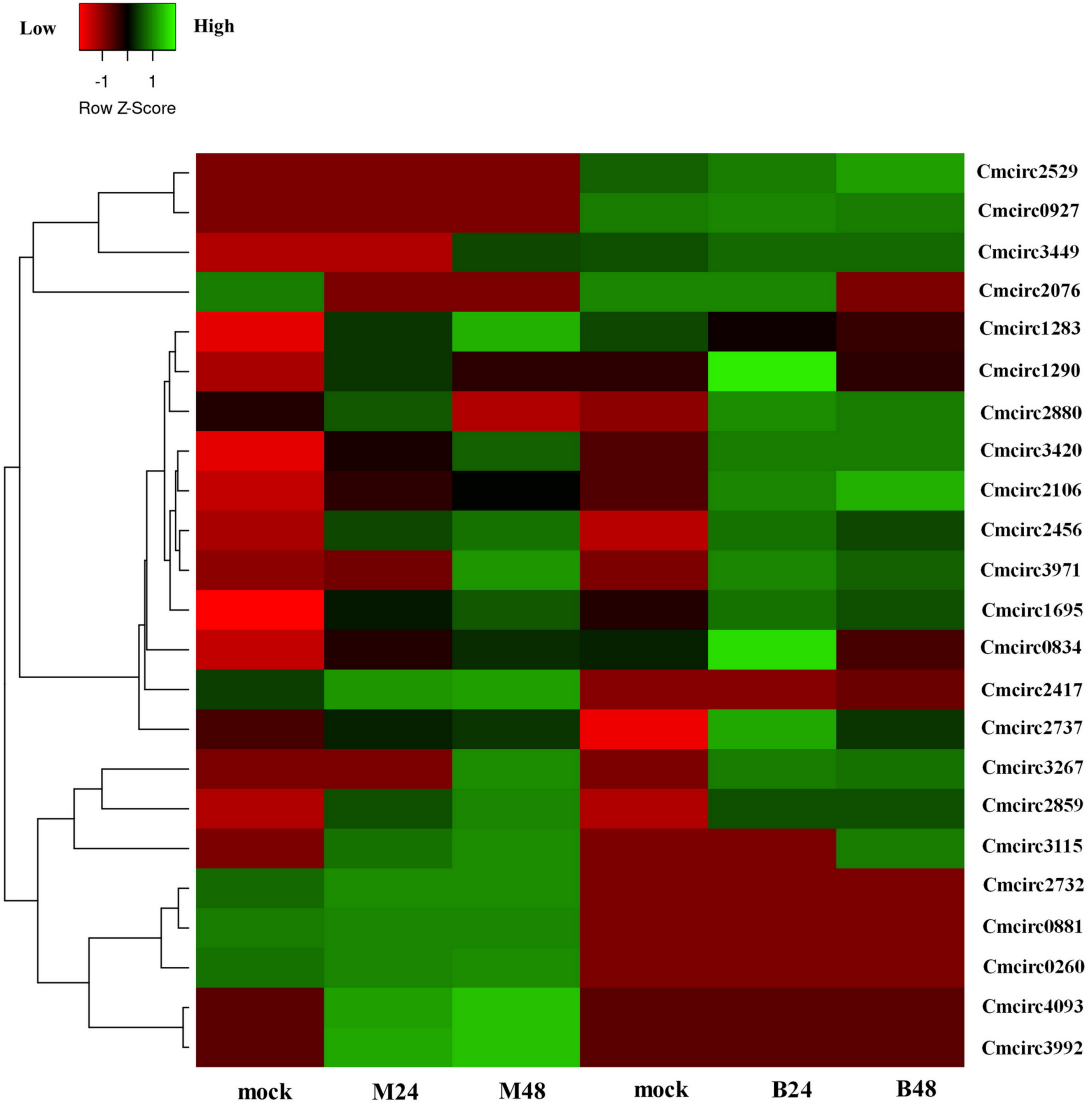
Clustering and differential expression patterns of 23 melon circRNAs after PM infection. The expression values were measured as TPM and presented as log2 (TPM value +1).

**Figure 6:**
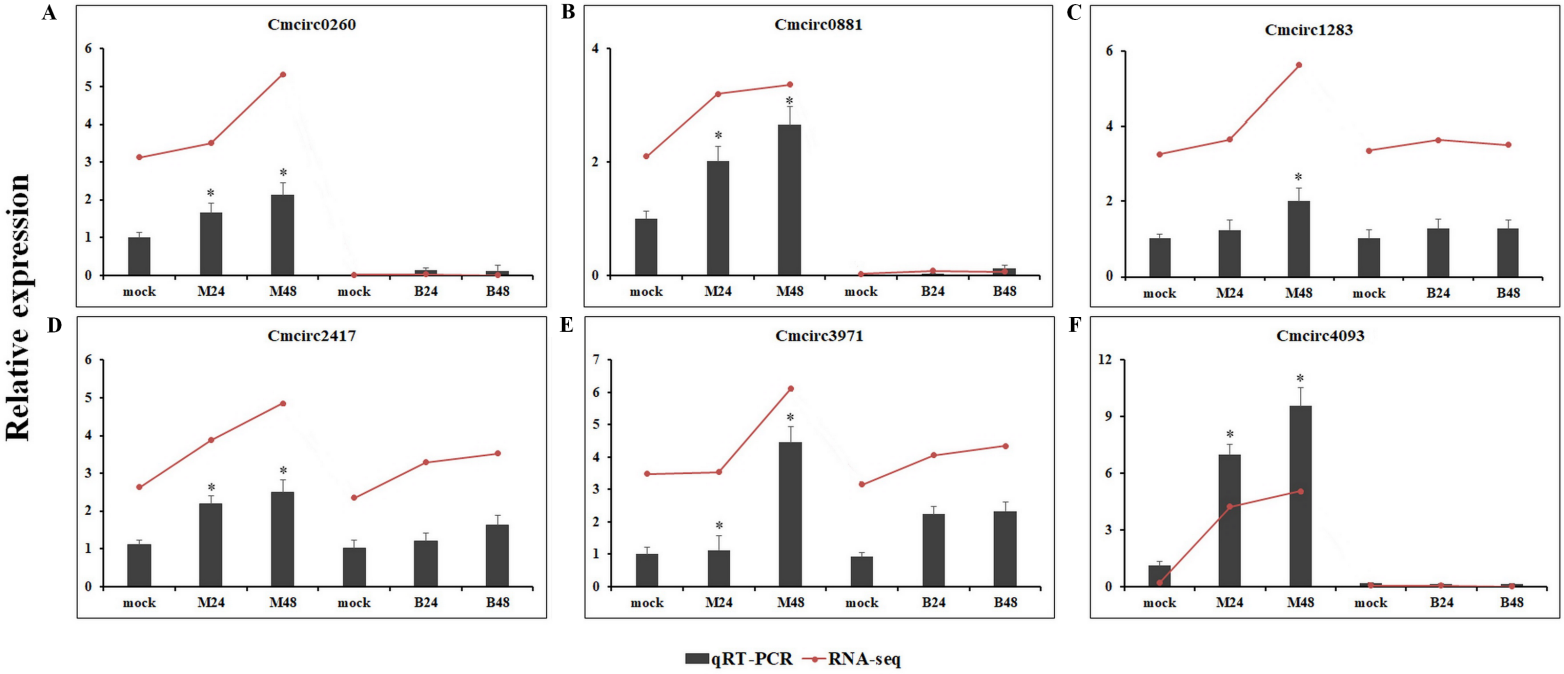
Experimental validation of six differential expressed circRNAs by qRT-PCR. (A) Cmcirc0260, (B) Cmcirc0881, (C) Cmcirc21283, (D) Cmcirc2417, (E) Cmcirc3971, (F) Cmcirc4093. *CmActin* was selected as internal reference. The relative expression quantity of circRNAs was normalized to that in mock. The red broken lines represent the RNA-seq values presented as log2 (TPM value + 1). Error bars indicate ± SD of three biological replicates. Asterisks indicated a significant change (^∗^*P* < 0.05; ^∗∗^*P* < 0.01) between different samples.

We quantified the expression of circRNAs in all samples to explore the biological function of melon circRNAs in response to PM infection and found that all melon circRNAs were expressed (TPM > 0) in at least one sample. Their differential expression profiles were evaluated between mock and PM infected samples, and a total of 13, 15, 13, and 13 circRNAs were found to be significantly up-regulated in M24, M48, B24, and B48, respectively. Only two, one, one, and two circRNAs were significantly down-regulated in B24, B48, M24, and M48, respectively (Figs. 4 and 5). Additionally, the expression levels of two and six circRNAs specifically changed in the PM-susceptible melon and PM-resistant melon, respectively (Fig. 4). Furthermore, qRT-PCR results confirmed that the expression changes of Cmcirc0260, Cmcirc0881, Cmcirc1283, Cmcirc2417, Cmcirc3971, and Cmcirc4093 in the PM-resistant melon were more noticeable than that in the PM-susceptible melon after PM infection, which was consistent with the transcriptome profiling results (Fig. 6).

### Functional characterization of parental genes of differentially expressed circRNAs

The molecular function of most circRNAs is not well defined, although some circRNAs have been shown to participate in the regulation of their parental gene expression. To explore the putative function of melon circRNAs, we identified the parental genes of melon circRNAs from the location where the circRNAs derived in the genome. One hundred and fifty-two parental genes were obtained for the 23 differentially expressed circRNAs and GO categories analyses were performed to explore their functions. The circRNA parental genes were mainly involved in the biological processes of gene expression, oxidation–reduction process, single-organism cellular process, response to biotic stimulus, and pathogenesis. Gene ontology of cellular components of parental genes showed that most of the gene products are cell parts, intracellular parts, membrane parts, and the macromolecular complex. The enriched GO terms for molecular function included catalytic activity, oxidoreductase activity, peroxidase activity, kinase activity, and protein binding (Table 1).

**Table 1:** GO enrichment analysis and functional categories of circRNA parental genes in melon.

GO_1accession	Functional category	Term type	*P*-value	Gene count
GO:0004096	catalase activity	molecular_function	2.28E−03	34
GO:0004601	peroxidase activity	molecular_function	5.30E−04	9
GO:0016684	Oxido-reductase activity	molecular_function	5.30E−04	6
GO:0005515	protein binding	molecular_function	6.62E−05	26
GO:0003824	kinase activity	molecular_function	9.01E−04	15
GO:0032991	macromolecular complex	cellular_component	6.99E−05	9
GO:0044425	membrane part	cellular_component	7.61E−04	11
GO:0044424	intracellular part	cellular_component	8.95E−05	5
GO:0044464	cell part	cellular_component	9.22E−04	17
GO:0009607	response to biotic stimulus	biological_process	8.62E−05	12
GO:0009405	pathogenesis	biological_process	1.14E−05	5
GO:0006952	defense response	biological_process	1.15E−04	6
GO:0055114	oxidation–reduction process	biological_process	2.18E−05	4
GO:0044710	single-organism metabolic process	biological_process	4.61E−04	9
GO:0010467	gene expression	biological_process	4.95E−05	5
GO:0044763	single-organism cellular process	biological_process	6.71E−04	18

### Identification of CircRNAs act as miRNA targets or ‘sponges’

circRNAs have been shown to interact with miRNAs to prevent them from targeting mRNAs and therefore may control target gene expression (Hansen et al., 2013). Binding between miRNA and circRNA is based on the complementarity between their nucleotide sequences. Many circRNAs can be cleaved by miRNA and were named as miRNA targets. However, many circRNAs can compete with mRNA to bind to specific miRNA without cleavage, thus blocking cleavage and alleviating the repression of its target mRNA. These circRNAs were called miRNA ‘sponges’. To identify circRNAs that may act as targets or ‘sponges’ of miRNA, we searched for the circRNAs and their complementary sequences against the melon miRNAs using the BLAST algorithm and found that 27 circRNAs can be bound by 18 miRNAs (Table 2). We predicted that some highly conserved miRNAs, such as miR156, miR159, miR161 and miR172, that had been functionally characterized, could be targeted by specific circRNAs. Of these circRNAs, some had more than one miRNA binding sites. For instance, Cmcirc03865 can be targeted by CmmiR419, CmmiR5021, and CmmiR5658. In addition, specific miRNA in melon could bind to different circRNA. For example, CmmiR5021 could bind with 11 different circRNAs and CmmiR5998 could bind to four different circRNAs.

**Table 2:** Predicted interaction between circRNAs and miRNA in melon.

CircRNA ID	miRNA ID	*E*-value	Sequence complementarity (%)
Cmcirc00352	CmmiR5021	2.15E−04	100
Cmcirc00352	CmmiR8170	6.36E−03	98.45
Cmcirc00422	CmmiR855	1.46E−04	90.28
Cmcirc00429	CmmiR865	4.80E−04	94.42
Cmcirc00464	CmmiR845	8.96E−04	100
Cmcirc00713	CmmiR5021	9.63E−05	93.24
Cmcirc00840	CmmiR447	9.85E−04	100
Cmcirc00916	CmmiR5021	1.59E−04	91.02
Cmcirc01282	CmmiR3434	4.56E−04	100
Cmcirc01283	CmmiR417	2.72E−04	88.95
Cmcirc01290	CmmiR161	1.48E−05	94.26
Cmcirc01351	CmmiR161	1.80E−04	89.95
Cmcirc01495	CmmiR5021	1.45E−05	96.66
Cmcirc01695	CmmiR5658	6.95E−04	89.69
Cmcirc01695	CmmiR5998	5.48E−04	87.58
Cmcirc01723	CmmiR156	5.70E−05	100
Cmcirc01723	CmmiR5658	2.00E−05	93.28
Cmcirc01736	CmmiR5021	2.37E−04	95.78
Cmcirc01736	CmmiR5658	8.70E−06	87.94
Cmcirc02030	CmmiR159	7.57E−06	100
Cmcirc02063	CmmiR5021	4.76E−04	95.21
Cmcirc02076	CmmiR5021	3.37E−06	90.14
Cmcirc02152	CmmiR5021	5.90E−04	90.55
Cmcirc02438	CmmiR5021	4.79E−04	88.56
Cmcirc02855	CmmiR5021	4.23E−05	97.84
Cmcirc02891	CmmiR417	4.79E−05	93.45
Cmcirc02905	CmmiR5633	1.78E−04	86.58
Cmcirc03115	CmmiR172	1.45E−03	100
Cmcirc03503	CmmiR779	6.67E−04	93.89
Cmcirc03756	CmmiR414	3.78E−04	97.25
Cmcirc03865	CmmiR419	4.00E−05	87.56
Cmcirc03865	CmmiR5021	8.89E−04	90.26
Cmcirc03865	CmmiR5658	4.78E−03	96.55

## Discussion

Recently, the identification and functional characterization of circRNAs has been widely reported in both animals and plants, demonstrating that circRNAs participate in multiple biological processes (Memczak et al., 2013; Chu et al., 2018). However, the molecular functions and regulatory mechanisms of circRNAs underlying PM resistance in melon is largely unknown. High-throughput sequencing and comparative transcriptome analysis were performed to identify putative circRNAs and their target genes involved in PM resistance. A total of 303 circRNAs were detected from the melon leaf. The number of circRNAs in melon was lower than in *Arabidopsis* (6,012), rice (12,037), cucumber (2,787), and soybean (5,372), which may be attributed to our more restrictive filter conditions for circRNA identification and the use of only leaf tissue for sequencing. Previous studies revealed that circRNA was expressed in a highly tissue- or stage-specific manner in most organisms (Tong et al., 2018).

It has been reported that circRNA can be generated from exons, introns, and intergenic regions of the genome and are named exonic circRNA, intronic circRNA, or intergenic circRNA, respectively (Zhang et al., 2013a; Zhang et al., 2013b; Jeck & Sharpless, 2014). In our study, exonic circRNAs were predominant (approximately 54.9%) compared to the intergenic (approximately 10%) and intronic (approximately 30%) circRNAs (Fig. 2). These proportions are similar to those in *Arabidopsis*, rice, cucumber, and tomato (Lu et al., 2015; Dou et al., 2017; Yin et al., 2018). In contrast, approximately 51% and 55% of the circRNAs were intergenic circRNAs in wheat and kiwifruit, which may be due to wheat’s large genome size with comparably fewer annotated genes (Wang et al., 2017a; Wang et al., 2017b). It has been deemed that the intergenic regions of a genome are more likely to contain missed genes that have not been annotated. Meanwhile, most of the total circRNAs were intronic circRNAs in soybean, which may be attributed to the genome duplication event responsible for generating multiple gene copies (Zhao et al., 2017). Our results indicated that the molecular basis of circular RNA biogenesis in plants varies among different species.

Differential expression analysis of circRNAs was conducted between mock and PM-inoculated samples of both PM-resistant and PM-susceptible genotypes. A total of 23 circRNAs were found to be responsive to PM infection, suggesting that these circRNAs may play important roles in PM resistance. RNA-seq data and qRT-PCR results confirmed that six circRNAs, including Cmcirc0260, Cmcirc0881, Cmcirc1283, Cmcirc2417, Cmcirc3971, and Cmcirc4093 in PM-resistant melon were more highly induced than that in PM-susceptible melon after PM infection, suggesting that a different disease response mechanism might exist between these melon genotypes. The functional annotation results showed that a large number of parental genes of circRNAs were involved in multiple redox processes such as the peroxidase gene and the glutathione reductase gene. Peroxidase and glutathione reductase play an essential role in the ROS scavenging pathway to prevent oxidative damage during the process of pathogen infection (Cui et al., 2017). The overexpression of a glutathione reductase gene (*SlGRE21*) prevented ROS accumulation and enhanced resistance against *P. infestans* in tomato (Cui et al., 2017). We also identified eight parental genes that encode pathogen-related proteins and the LRR receptor, implying that circRNAs could regulate the gene expression to resist against PM infection in melon.

Previous reports have shown that circRNAs can regulate gene expression in a miRNA-mediated manner. They may act as targets or ‘sponges’ of miRNAs to restrict the cleavage of target genes mediated by miRNA and thus promote target gene expression (Hansen et al., 2013). Similar to the results in *Arabidopsis*, cotton, and other plants, many melon circRNAs are thought to be miRNA ‘sponges’. Twenty-seven circRNAs were predicted to be potential targets of 18 family miRNAs including miR159, miR161, miR417, miR419, miR845, miR5021, and others. A few miRNAs including miR845, miR855, miR5021, and miR5658 were confirmed to be involved in the response to various biotic and abiotic stresses (Borges et al., 2018). Our results suggested that direct interactions between miRNAs and circRNAs may also exist in melon, providing new information for further investigation into the function and mechanisms of circRNAs in PM resistance.

## Conclusions

Our study was the first to report the existence of abundant circRNAs in the melon leaf and to characterize their possible regulatory roles in response to PM disease. Nevertheless, it should be noted that only leaf tissue was included in our study, which may limit the amount and properties of circRNAs in melon that we were able to find. Future studies should be conducted to detect circRNAs from more tissues and various developmental stages and to determine the mechanism of melon circRNAs in resisting PM. Our findings improved the understanding of circRNAs in *cucurbitaceae* species and provided new insights into the regulatory roles of circRNAs in PM resistance.

##  Supplemental Information

10.7717/peerj.11216/supp-1 Supplemental Information 1 Length distribution of all circRNAs identified in melon

10.7717/peerj.11216/supp-2 Supplemental Information 2 Primer sequences used for circRNA validation and qRT-PCR

10.7717/peerj.11216/supp-3 Supplemental Information 3 The detailed information of RNA-seq data and mapping results

10.7717/peerj.11216/supp-4 Supplemental Information 4 The Cycle threshold (Ct) values used for qRT-PCR analysis

10.7717/peerj.11216/supp-5 Supplemental Information 5 The sequence of the back-spliced junction sites corresponding to the circRNAs that have differential abundance

## Additional Information and Declarations

### DNA Deposition

The following information was supplied regarding the deposition of DNA sequences: The raw data are available in the NCBI Sequence Read Archive database: SRR9129105–SRR9129122.

### Data Availability

The following information was supplied regarding data availability: The raw measurements are available in the Supplementary Files.
