# Supplementary figures and images for: Identification and characterization of melon circular RNAs involved in powdery mildew responses through comparative transcriptome analysis
Source: PeerJ. 2021 Apr 15;9:e11216. doi: 10.7717/peerj.11216 (PMC8053381; doi:10.7717/peerj.11216)

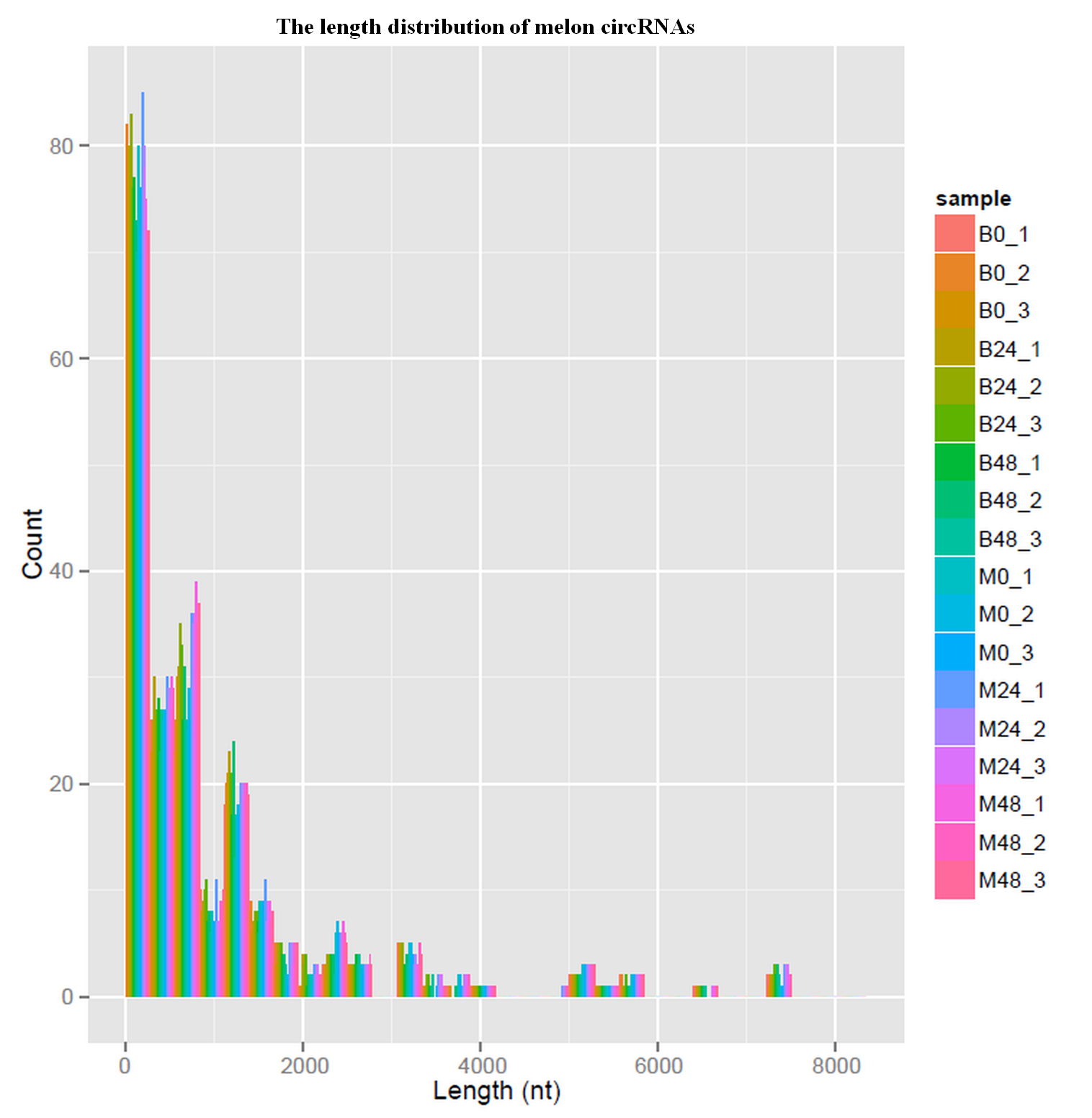

Supplement: Supplemental Information 1 [file peerj-09-11216-s001.png]
