# Supplementary material for: Identification and characterization of melon circular RNAs involved in powdery mildew responses through comparative transcriptome analysis
Source: PeerJ. 2021 Apr 15;9:e11216. doi: 10.7717/peerj.11216 (PMC8053381; doi:10.7717/peerj.11216)
Supplement: Supplemental Information 3 [file peerj-09-11216-s003.docx]

Supplementary Table S2 The detailed information of RNA-seq data and mapping results

| Sample name | Raw reads | Clean reads | Total mapped unique reads (%) | Junction reads | CircRNA amount |
| --- | --- | --- | --- | --- | --- |
| B0_1 | 118010586 | 115800212 | 85.26 | 2305 | 190 |
| B0_2 | 111356468 | 106406988 | 86.30 | 2114 | 193 |
| B0_3 | 126911056 | 120942420 | 85.07 | 2447 | 197 |
| B24_1 | 105996486 | 99074280 | 86.31 | 2014 | 212 |
| B24_2 | 115865820 | 109081240 | 86.07 | 2096 | 215 |
| B24_3 | 122783160 | 119067176 | 86.27 | 2289 | 208 |
| B48_1 | 114601256 | 114314170 | 86.52 | 2158 | 188 |
| B48_2 | 115301178 | 107675836 | 84.45 | 2011 | 193 |
| B48_3 | 108250146 | 94267112 | 83.12 | 1992 | 174 |
| M0_1 | 117713532 | 100535446 | 83.59 | 2001 | 194 |
| M0_2 | 108293262 | 95155226 | 84.86 | 1997 | 190 |
| M0_3 | 103308482 | 99862912 | 85.09 | 1896 | 186 |
| M24_1 | 100734002 | 98223418 | 85.92 | 1845 | 227 |
| M24_2 | 100841694 | 98135728 | 84.87 | 1942 | 213 |
| M24_3 | 101153390 | 95676696 | 84.86 | 1931 | 209 |
| M48_1 | 116168260 | 106591602 | 84.53 | 2541 | 214 |
| M48_2 | 105834776 | 97652272 | 84.74 | 1994 | 204 |
| M48_3 | 110926652 | 106440110 | 85.42 | 2215 | 207 |
